# Feasibility of a virtual reality course on adult tracheostomy safety skills*

**DOI:** 10.1002/anr3.12305

**Published:** 2024-06-17

**Authors:** J. R. Abbas, E. Bertram‐Ralph, S. Hatton, T. Garth, C. Doherty, I. A. Bruce, B. A. McGrath

**Affiliations:** ^1^ Faculty of Biology Medicine and Human Health The University of Manchester Manchester UK; ^2^ Human Factors Academy, Manchester University NHS Foundation Trust Manchester UK; ^3^ Acute Intensive Care Unit, Wythenshawe Hospital Manchester University NHS Foundation Trust Manchester UK; ^4^ North West School of Anaesthesia Manchester UK; ^5^ Department of Anaesthesia Manchester University NHS Foundation Trust Manchester UK; ^6^ Department of Paediatric Otorhinolaryngology, Royal Manchester Children's Hospital Manchester University NHS Foundation Trust Manchester UK; ^7^ Acute Intensive Care Unit, Wythenshawe Hospital Manchester University NHS Foundation Trust Manchester UK

**Keywords:** emergency skills training, tracheostomy, virtual reality simulation

## Abstract

The National Tracheostomy Safety Project has run high‐quality, face‐to‐face skills courses since 2009. The aim of this project was to produce a virtual reality version of the established course and evaluate its impact on participant learning, and participant and faculty satisfaction. Healthcare staff and students were recruited and randomised to attend one of (1) a face‐to‐face traditional course (control); (2) a virtual reality course at a conference centre with on‐site technical support; (3) a fully remote virtual reality course; the virtual reality groups were combined for the analysis of learning outcomes and satisfaction. The primary outcome was the difference in pre/post‐course knowledge scores on a 30‐item questionnaire; secondary outcomes included knowledge retention, usability, comfort/side effects and participant performance in a simulated tracheostomy emergency. Thirty‐seven participants and 15 faculty participated in this study. There was no significant difference between mean pre/post‐course scores from the face‐to‐face (from 21.1 to 23.1; +2) and combined virtual reality (from 17.1 to 21.1; +4) groups, with both showing improvement (p = 0.21). The mean System Usability Scale score for virtual reality was 76.8 (SD 12.6), which is above average; the median Simulator Sickness Questionnaire score was 7.5 (IQR 3.7–22.4), indicating minimal symptoms. All participants resolved the primary clinical problem in the simulated emergency, but the virtual reality (VR) group was slower overall (mean difference 61.8 s, p = 0.003). This technical feasibility study demonstrated that there was no difference in participant knowledge immediately after and 4 weeks following face‐to‐face and virtual reality courses. Virtual reality offers an immersive experience that can be delivered remotely and offers potential benefits of reducing travel and venue costs for attendees, therefore increasing the flexibility of training opportunities.

## Introduction

As virtual reality (VR) technology continues to develop, it is predicted to significantly impact medical education, patient outcomes and staff and student well‐being [1]. The technology is advancing rapidly, and although the uptake in the consumer market looks promising, it is essential to clarify whether VR can be used to replace elements of traditional learning [2].

Patients undergoing tracheostomy require high‐quality interprofessional care to maintain safety and avoid serious clinical incidents, which occur in around 30% of patients [3, 4]. Landmark studies have demonstrated that quality training can lead to avoidance of these potentially fatal complications [5]. Whilst having the potential to deliver such training, VR may in addition overcome some well‐described limitations of conventional pedagogical approaches, such as high cost, consistency of experience, the need for specialised venues to run sessions and the high carbon footprint of travel, equipment and consumables [6, 7].

By measuring knowledge gain and retention, participant performance in simulation, acceptability, adverse effects and participant and trainer satisfaction, this study used multiple methods to evaluate the technical feasibility of a virtualised version of a long‐running adult tracheostomy safety course.

## Methods

An expert steering committee developed and evaluated the VR course and included senior clinicians, researchers, educationalists and a VR development team. Both the VR and face‐to‐face (F2F) courses in this study were designed to align with best practices in simulation standards [8].

### Study setting, ethics and participants

This study took place within Manchester University NHS Foundation Trust. Ethical review (2023‐16428‐28024) was obtained and written consent was collected from each participant. Inclusion criteria were healthcare staff or students who were required to train and maintain skills in tracheostomy management. Participants were excluded if they only treated children or never cared for patients living with a tracheostomy.

Participants declared their availability for any of the three proposed course dates, but the nature of the courses (F2F or VR) were not disclosed at registration. After considering availability, participants were randomly allocated to one of three methodological arms of the study using RStudio v2023.06.0+421 (RStudio, PBC, Boston, MA, USA). These represented three‐course variants: F2F (face‐to‐face control), VR‐F2F (intervention; VR course with F2F faculty and technical support available) and VR‐Remote (intervention; fully remote VR course with remote faculty and technical support).

Course delivery mirrored the established National Tracheostomy Safety Project (NTSP, www.tracheostomy.org.uk)/Advanced Life Support Group (ALSG, www.alsg.org) courses, detailed in Table 1. The VR course content was presented to the user with instructions on a tablet computer paired with a VR headset (Pico Neo‐3 Pro, Pico Technology, Cambridgeshire, UK).

**Table 1:** Detailed outline of the course components and delivery differences between study arms.

Course component	Detail
**Introduction** **(lecture format)**	Welcome, aims and objectives of education delivery Research overview. Fire safety and housekeeping for face‐to‐face arms of study *For both VR arms – opportunity to familiarise with VR hardware* *For remote VR arm – delivery was via Zoom teleconferencing software*
**Algorithm demonstration** **(video or live demonstration)**	Perfect run‐through of response to tracheostomy emergency. This allowed familiarisation for simulation (virtual and live mannequin‐based demonstration) *For VR demonstration 360‐degree videos and perfect VR simulation run‐throughs was displayed in headset*
**Skills station 1: Airway** **(interactive design)**	Discussion around importance of ventilation in the delivery of oxygen. Instructors demonstrated basic and advanced airway techniques with an opportunity to practice these *For both VR components the demonstration was delivered in 360 video*
**Skills station 2: Red flags** **(small group discussion in a multi‐user VR environment)**	Delivery of a PowerPoint presentation with videos supported by live faculty *For both VR components this is a guided discussion with breaks for questions*
**Skills station 3: Equipment** **(small group discussion in a multi‐user VR environment)**	Introduction to the equipment required to deliver basic nursing care to ensure patient safety *For both VR components the equipment is provided in a “sand box” environment where participants could pick up and interact with the equipment in VR*
**Skills station 4: Emergency training** **(VR or low‐fidelity manikin small group simulation)**	Emergency scenarios were presented to candidates and their performance was evaluated. A faculty‐led debrief occurred after each simulation *For both VR components participants were presented with near‐identical scenarios to the face‐to‐face course in a group simulation which included up to five participants and a faculty observer. Debriefs occurred in a dedicated multi‐user VR meeting space after each simulation*
**Close** **(lecture format)**	Summary, overview of course and opportunity to answer any participant questions and close *For remote VR components, questions were taken in the smaller groups of 5 participants at the end of the simulation session. The final closing lecture follows with a recap, clinical relevance and summary*

VR, virtual reality.

### Outcomes and outcome measure instruments

The primary outcomes were knowledge gain and retention which were measured using an online knowledge test adapted from the validated NTSP e‐Learning for Healthcare modules (www.tracheostomy.org.uk/e‐learning), delivered in a pre‐ and post‐course intervention approach and repeated at 4 weeks following the course (Supporting Information, Appendix S1).

Secondary outcomes included VR usability, comfort/adverse effects from participation, course evaluation (participants and facilitators) and emergency management skill performance. Virtual reality usability and comfort/side effects were measured using the validated System Usability Scale (SUS) and Simulator Sickness Questionnaire (SSQ), respectively [9, 10]. Participant and faculty satisfaction were measured using a bespoke post‐course evaluation questionnaire (Supporting Information, Appendix S2). Participant performance managing a tracheostomy emergency was measured using simulation sessions in VR during the VR arm of the study and during traditional manikin simulation sessions for the control arm of the study. The participants being evaluated were asked to manage the simulated emergency from the perspective of a first responder, whereas other participants were asked to play the role of assisting clinical team members. Due to course time constraints, not all participants were able to be assessed as a first responder. The specific roles for each scenario were assigned during the sessions on a voluntary basis. All simulation sessions were recorded and were later reviewed by the authors; the times to perform critical interventions were noted. A brief, high level cost analysis is included, but full economic evaluation is beyond the scope of this work.

### Statistical analysis

Data distributions were examined with box and whisker plots and Shapiro–Wilk tests using SPSS Statistics 29.0 (IBM Corp, Armonk, NY, USA) and reported as mean (standard deviation) or median (interquartile range), as appropriate. Differences in knowledge scores were evaluated at three time points using one‐way repeated measures analysis of variance (ANOVA). Chi‐square tests were used for group comparisons in evaluation outcomes. Both VR intervention groups were combined for the All‐VR analysis providing the main results of this study. The decision to split the intervention arm into the two groups (VR‐F2F and VR‐Remote) was primarily pragmatic. It was a priority that during the first VR iteration (VR‐F2F) of this course, participants had direct access to the technical expertise that may be required prior to attempting a fully virtual (VR‐Remote) iteration. This study was intended to provide pilot data and, in part, to provide insights as to how much support the participants require from a technological perspective. As such, most of the analysis of outcomes groups the two VR groups together (All‐VR), although some sub‐group analysis between the VR‐F2F and VR‐Remote groups has also been performed. Significance was assumed at p < 0.05.

## Results

The three courses ran between 26 April and 5 May 2023. Each course lasted 4 h. A total of 66 participants were recruited, in addition to 15 faculty. The same group of faculty supported all three course iterations. Additionally, two authors (JRA and BAM) facilitated the course days, but did not complete any data collection tools. After randomisation, 29 registered participants could not attend their allocated course and were withdrawn from the study, leaving a total of 37 participants enrolled. Sixteen participants undertook the F2F (control) course, 10 undertook the VR‐F2F course and 11 undertook the VR‐Remote course (totalling 21 candidates in the All‐VR intervention arm). Participants were from varied clinical backgrounds including 13 doctors, 16 nurses, 6 physiotherapists, 1 educationalist and 1 speech and language therapist. All participants completed all of the data collection tools.

### Knowledge

No significant outliers were identified. The pre‐ and post‐course knowledge test scores demonstrated that the intervention group had a lower baseline level of knowledge than the control group (mean scores of 17.1(3.7) vs 21.1(3.2), respectively, p < 0.01). Across all participants in all arms of the study, knowledge scores increased following the course (p < 0.01, partial η^2^ 0.285). Knowledge scores increased from pre‐course to post‐course (mean difference 3.2, 95% CI 1.2–5.1, p < 0.01) and did not reduce significantly over the time (4 weeks) between post‐course and retention tests (mean difference 0.0, 95% CI −1.2–1.2, p = 1.0).

**Figure 1:**
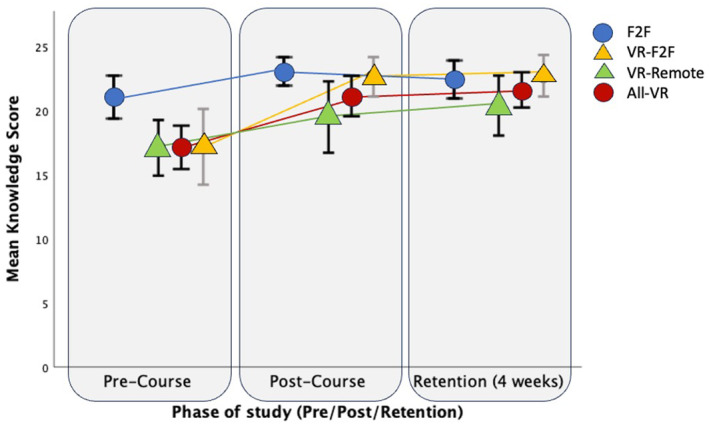
Mean knowledge score, clustered by phase of study (pre‐course, post‐course or retention) and grouped by type of course attended. Bars represent 95% confidence intervals. F2F, face‐to‐face; VR, virtual reality.

There was a significant increase in the mean improvement between pre‐ and post‐knowledge scores in the intervention group (All‐VR) from 17.1 to 21.1 (+4, p < 0.01). There was a non‐significant improvement in the control (F2F) group from 21.1 to 23.1 (+2, p = 0.053). This difference in the pre‐post score improvement between the two groups was not significant (p = 0.21). Figure 1 demonstrates the changes in knowledge test scores throughout all study phases, grouped by course allocation. Knowledge gain is evident in the combined cohort, with knowledge retained at 4 weeks (p < 0.01, partial η^2^ 0.285).

### Usability, comfort and side effects

The All‐VR group had a mean SUS score of 76.8 (12.6), considered above average, with the VR‐F2F group scoring 70.2 (10.2) and the VR‐Remote group scoring a significantly (p < 0.05) higher score of 82.7 (11.9). The median total SSQ scores combining all domains (nausea, oculomotor and disorientation) was 7.5 (3.7–22.4) in the All‐VR group, which is considered minimal. In the VR‐F2F group the SSQ was 11.2 (4.7–17.8), and VR‐Remote group was 7.48 (3.7–22.4). This difference did not reach statistical significance.

### Performance

In the F2F group, 15 participants completed the assessment as primary responders; in the combined VR groups, 10 were evaluated. All participants completed the scenario successfully. Table 2 summarises the time to critical specific interventions during the simulation. The VR group were significantly slower to achieve all critical interventions than the F2F groups except for the application of oxygen (p = 0.127). The mean difference in completing the scenario was 61.8 s, favouring F2F group performance over the performance assessed in the All‐VR group (p = 0.003).

**Table 2:** Summary of performance metrics across intervention and control groups. Time to key interventions in simulation are shown in seconds alongside the differences between groups. In the F2F group, 15 participants completed the assessment as primary responders; in the intervention groups, 10 were evaluated.

Time to key intervention	Intervention or control	Mean (SD) (seconds)	Mean difference (seconds)	p Value
**Call for help**	Intervention	35.2 (24.3)	24.4	0.011
	Control	10.8 (6.8)		
**Apply oxygen**	Intervention	68.5 (24.1)	15.9	0.127
	Control	52.6 (25.1)		
**Resolve primary issue (decannulation)**	Intervention	194.3 (43.0)	56.9	0.002
	Control	137.4 (25.6)		
**Total scenario time**	Intervention	227.4 (48.2)	61.8	0.003
	Control	165.6 (31.4)		

### Participant (learner) satisfaction

Twenty participants in the All‐VR group rated the course as ‘excellent’, with one candidate rating the course overall as ‘good’. Fourteen of the F2F control group rated the educational experience as ‘excellent’, with two rating it as ‘good’. There was no difference between the VR and F2F groups (Chi^2^ p = 0.735). Virtual Reality (VR) group candidates were also asked, ‘From the VR training experience, do you feel VR has the potential to impact patient and team safety and quality?’ Eighteen participants responded ‘yes’, and three responded ‘maybe’. There was no discernible difference in participant satisfaction between VR candidates who took part remotely or F2F.

### Participant (faculty) satisfaction

Twelve out of 15 faculty completed post‐course questionnaires. Seven rated the intervention as ‘excellent’, and five concluded that the overall experience was ‘good’. When asked the question, ‘From the VR training experience, do you feel VR has the potential to impact patient and team safety and quality?’ Eleven participants responded, ‘yes’ and one answered ‘maybe’. All Likert responses assessing satisfaction across all course areas were either ‘agree’ or ‘strongly agree’.

### Cost analysis

When considering the headline economic costs of hosting an F2F or VR course, there are several one‐off and recurrent costs that need to be accounted for. The capital costs included the cost of simulation manikins (we had five Tracheostomy Trainer Manikins totalling £12,475), other supporting simulation equipment (mostly out of date stock, £0), and VR headsets (Pico Neo‐3 retailing at approximately £595 × 15, totalling (£8925)). The value of these items is expected to depreciate over time. The VR headsets may additionally be used for other applications and, with every subsequent course, be cheaper per use. We were unable to obtain accurate costs for the infrastructure of the F2F venue, but this is factored into the hire costs of £1000 per day. The fully remote courses incurred the costs of re‐usable protective cases at £295 × 10 (£2950). Both versions of the course required the same number of instructors, but travel costs were significantly reduced with the virtual courses for both faculty and candidates. Whilst we did not set out to collect comprehensive data to evaluate the economic costs of these courses, the initial set‐up costs are broadly similar. However, it is likely that the recurrent costs and the carbon footprint (travel) of the virtual courses are less than the F2F course [6].

## Discussion

This study demonstrates that an adult tracheostomy safety course may be delivered in VR, producing similar educational outcomes to traditional F2F courses. Participants and faculty found the VR experience valuable and enjoyable, and we did not record any significant adverse effects among participants. Virtual reality may be a viable alternative to traditional F2F simulation skills training in the context of tracheostomy education.

Emergency algorithm training is an area of particular focus for the NTSP when delivering this national course [4]. Emergency scenario education is a common focus for VR educational development, and a range of novel learning tools have been established with positive educational outcomes [11]. We used a relatively low‐fidelity VR system to deliver identical and explicitly stated learning outcomes to the long‐established NTSP adult tracheostomy safety course. All of the learning outcomes were assessed through the above‐described outcome measure instruments.

Although participants completed the assessment satisfactorily, slower performance was seen in VR. We cannot conclude that this is an inherent failing in the VR solution as it is logical to assume that inexperience using VR may have slowed progress. It remains to be seen whether this would translate to any difference in clinical care.

Current literature suggests that VR content focusing on decision‐making, equipment recognition and algorithmic learning is likely to achieve positive educational results, as reflected here [12, 13]. Using VR to train the fine motor skills of technical procedures is possible; however, capable systems are considerably more expensive [14]. The approach taken in this study to split the VR participants into groups who participated in VR with F2F support or entirely remotely allowed the research and education team to first gain familiarity with the hardware, software and deliver a course using this technology. Several lessons were learnt during the VR‐F2F arm of the study. Firstly, the research team were able to troubleshoot common issues around internet connectivity and software bugs, which required a reboot of the system to rectify them. Secondly, the majority of the faculty were entirely new to VR, and this gave them an opportunity to learn how to adjust headsets for comfort and optimum experience. Lastly, authors JA and BAM, alongside technical support teams were available to immediately assist, further educating the faculty. This facilitated a solid understanding of the system and solutions to frequently encountered technical problems in advance of the fully remote iteration.

Miller described the pyramid of clinical competence in 1990 and classified competence into four levels (‘knows’, ‘knows how’, ‘shows how’ and ‘does’) [15]. Simulation assessment, as utilised in both arms of this study, tested whether the user ‘shows how’ a tracheostomy emergency is managed. Considering the immediate and retained knowledge gain that this study demonstrates (‘knows’), we have shown that both VR and face‐to‐face course variants may lead to adequate clinical knowledge acquisition. Interestingly, the baseline level of knowledge differed between groups. This may have been due to an imbalance in professional background or seniority represented within these groups. Baseline knowledge may have been equivalent in subset analyses isolating professional groups; however, this was outside the remit of the presented aims of the study. Previous work by the NTSP has shown that patient safety can be improved significantly by training staff using NTSP resources and face‐to‐face courses [5]. However, translation from VR course participation into patient safety benefits is yet to be demonstrated.

As educators and institutions look towards technologies to widen access to healthcare education, educational validation must not be the only academic focus [16]. To realise the potential benefits of technology‐enhanced education, systems must be comfortable, usable and have a perceived ‘relative advantage’ over existing tools [17, 18]. Our study demonstrated that both faculty and learners found the experience positive, without significant side effects. Interestingly, participants who attended the VR course remotely found the system significantly more usable than those who attended on‐site. No data were collected to explain this unexpected finding, and the hardware and software were identical between courses, with no changes to the VR environment or course content. One can postulate that doing a VR course in a participant's chosen environment may be preferable, perhaps due to not being directly observed as they attempt to navigate a novel technology. Further work is required to explore this interesting point. The wide ranging SSQ scores may indicate that for some individuals, this VR system is not acceptable, but overall, participants rated the VR course as ‘excellent’, the majority of whom also acknowledged the wider potential educational benefit of VR.

Our study has several limitations. The sample size was small with an uneven intervention to control group ratio and insufficiently powered for non‐inferiority calculations. Additionally, participants were unmatched, with broad clinical backgrounds. Whilst this may lead to broader applicability of the results, it was not intended to discover differences within or between professional groups. Further to this, non‐concealed allocation may have led to bias as specific individual characteristics may be more likely to take part, such as participants' innate interest in simulation skills training, technology‐enhanced learning or educational research. Lastly, as we combined data from the two implementations of VR training, we acknowledge the inherent differences in the delivery and the unknown effect that may have had on outcomes. Our data indicate similar or even favourable experiences for a fully remote course, which will be the focus of future work.

Faculty and learners reported technology limitations such as programming bugs, reboot following crashes and internet connection difficulties, which proved prohibitive for one participant. Despite the manufacturer's descriptions, the headset's battery life was insufficient to run an entire educational session on a single battery charge. We recommend that mobile power banks be provided as standard for future course iterations. Solving these issues is essential to the consistent delivery of quality VR education. Throughout both VR courses, we relied on access to fast internet connections, at least sufficient to stream ultra high definition video. In order to minimise the impact of concurrent users on available bandwidth, we staggered the timetable of the VR activities.

VR education has well‐documented limitations, but if content is designed carefully to build on what VR does well, effective knowledge and skills acquisition and retention are possible, with potential advantages for learners and trainers. We welcome additional research in this emerging space.

## Supporting information


**Appendix S1.** Knowledge Questionnaire.

**Appendix S2.** Course Evaluation Questionnaire.
