# Supplementary material for: Feasibility of a virtual reality course on adult tracheostomy safety skills*
Source: Anaesth Rep. 2024 Jun 17;12(1):e12305. doi: 10.1002/anr3.12305 (PMC11182690; doi:10.1002/anr3.12305)
Supplement: Supplementary file 1 — Appendix S1. Knowledge Questionnaire. [file ANR3-12-e12305-s001.docx]

**Abbas et al; Supplemental Material 1.** Knowledge Questionnaire

1. In an emergency, oxygen can be delivered to a laryngectomee using a facemask applied to the mouth.
   1. True
   2. False
2. Which of the following are recognised indications for a temporary tracheostomy? True or False for each.
   1. Inability to protect the airway after a head injury
   2. Severe facial trauma
   3. Surgical removal of carcinoma of the larynx
   4. Weaning from mechanical ventilation
   5. Inability to clear secretions
3. The laryngectomy emergency algorithm is very similar to the tracheostomy algorithm, however, there are a few key differences. Considering the anatomical and physiological differences, would you expect to see the following elements in the laryngectomy algorithm? True or False for each.
   1. Apply oxygen to the face and stoma
   2. Prepare for difficult oral intubation
   3. Remove speaking valve (if present)
   4. Consider the use of a bougie/aintree catheter
   5. Consider the use of an endoscope.
4. Which of the following statements are appropriate initial interventions for a deteriorating patient who is NOT breathing, following removal of a blocked tracheostomy. True or False for each.
5. Apply chest compressions
6. Intubate the stoma with a 6.0 mm endotracheal tube
7. Intubate the stoma with a new 7.0 cuffed tracheostomy tube
8. Insert an oral Supraglottic Airway Device (SAD)
9. Apply a SAD to the stoma and attempting to ventilate
10. The following situations could be considered tracheostomy 'Red Flags'. True or False for each.
    1. Suction catheter only passes intermittently
    2. Patient has an uncuffed tube in situ, breathing spontaneously via trachy-mask, and can talk softly
    3. 60 mL of air is required to inflate the tracheostomy tube cuff
    4. Blood-stained sputum has been produced overnight following a percutaneous tracheostomy the previous day
    5. Bubbling from the mouth of a patient ventilated via a cuffed tracheostomy
11. The presence of a tracheostomy tube is a contraindication for eating and drinking particularly in the critically ill patient.
    1. True
    2. False
12. It is impossible for a patient to vocalise following a total laryngectomy.
    1. True
    2. False
13. Which of the following statements concerning secondary emergency oxygenation are NOT correct?
    1. An Aintree catheter can be loaded onto an endoscope and used to ensure that a new tube is relocated into the trachea
    2. An uncuffed endotracheal tube can be advanced orally beyond the stoma to 'seal off' any leak from the stoma when positive pressure ventilation is applied
    3. An Aintree catheter can be used with waveform capnography
    4. Digital manipulation of a bougie into the trachea is a recognised technique when reinsertion is difficult
    5. An emergency cricothyroidotomy can be useful, even if the patient has a tracheostomy
14. Getting patients with tracheostomies talking again can have a positive impact on anxiety and depression.
    1. True
    2. False
15. In which of these situations would you only get airflow through the tracheostomy tube or stoma, and not the upper airway (nose and mouth)? True or False for each.
16. Patient with an uncuffed, fenestrated outer tube with an unfenestrated inner tube in situ
17. Patient following a total laryngectomy
18. Patient with an cuffed tracheostomy tube (inflated)
19. Patient with a cuffed tracheostomy tube (inflated) and speaking valve
20. Patient with a fenestrated, uncuffed tube and speaking valve.
