# Supplementary material for: Feasibility of a virtual reality course on adult tracheostomy safety skills*
Source: Anaesth Rep. 2024 Jun 17;12(1):e12305. doi: 10.1002/anr3.12305 (PMC11182690; doi:10.1002/anr3.12305)
Supplement: Supplementary file 2 — Appendix S2. Course Evaluation Questionnaire. [file ANR3-12-e12305-s002.docx]

**Abbas et al; Supplemental Material 2.** Course Evaluation Questionnaire

1. How did you find the orientation to the VR session?  Single choice.

- Excellent
- Very Good
- Good
- Adequate
- Required more information

1. Please add any additional comments/suggestions for Question 4 below.

Enter your answer

1. The introduction was useful. Single choice.

- Strongly agree
- Agree
- Neutral
- Disagree
- Strongly disagree

1. The session teaching red flags was useful. Single choice.
   - Strongly agree
   - Agree
   - Neutral
   - Disagree
   - Strongly disagree
2. The session teaching equipment was useful. Single choice.

- Strongly agree
- Agree
- Neutral
- Disagree
- Strongly disagree

1. The session teaching airway skills was useful. Single choice.

- Strongly agree
- Agree
- Neutral
- Disagree
- Strongly disagree

1. The session demonstrating a tracheostomy emergency was useful. Single choice.

- Strongly agree
- Agree
- Neutral
- Disagree
- Strongly disagree

1. The practical tracheostomy emergency was useful. Single choice.

- Strongly agree
- Agree
- Neutral
- Disagree
- Strongly disagree

1. From the VR training experience do you feel VR has the potential to impact patient and team safety and quality?  Single choice.

- Yes
- No
- Maybe

1. Please add your thoughts regarding the potential impact to patient and team safety below.

Enter your answer

1. Once you are confident with the system what would be your preferred locations/s for VR training?

- VR headsets located on wards / within critical care
- VR headsets set up in classrooms / sim environments
- VR headsets personally allocated for home use
- VR headsets in break areas
- VR headsets that can be booked through a library or education department

1. How would you feel about being able to take a VR headset home and completing the training remotely?

- Very comfortable
- Somewhat comfortable
- Somewhat uncomfortable
- Very uncomfortable
- Neither comfortable nor uncomfortable

1. Please add any additional comments regarding VR home based learning below.

Enter your answer

1. Do you feel VR training is an efficient and effective education modality for healthcare staff/teams? Single choice.

- Yes
- No
- Maybe

15. Comments regarding question 14.

Enter your answer

16. What would be your overall rating of this VR educational experience.

- 1- very poor
- 2
- 3
- 4
- 5- excellent

17. Please add any additional suggestions or ways the team could improve the system or training experience. Thank you for taking the time to complete this survey.

Enter your answer
